# Increased calf and plantar muscle fibrotic contents in obese subjects may cause ankle instability

**DOI:** 10.1042/BSR20160206

**Published:** 2016-08-16

**Authors:** Junwei Zhu, Lei Zhang, Yong Chen, Jianning Zhao

**Affiliations:** * Department of Orthopedics, Jinling Hospital, Nanjing University School of Medicine, Nanjing 210002, China

**Keywords:** ankle, BMI, joint loading, MRI, stability

## Abstract

Obesity is strongly associated with musculoskeletal disorders of the lower limb, including ankle instability and resulting gait problems. In the present study, we aimed to examine, using paired comparisons of subjects, whether moderate duration of obesity in patients with mild to moderate elevations of body mass index (BMI), changes the fibrous contents of muscles that support the ankle mortices, namely calf and plantar muscles. We attempted to examine these parameters because this shall provide direct evidence of whether obesity directly impacts myoarchitecture and support of the adjoining joints. MRI image segmentation and pixel correlations by grey level co-occurrence matrix (GLCM) and entropy were used to analyse the changes. The differences in the means between groups (both GLCM and entropy) were significant from control lean populations (*P*<0.0001, ANOVA) for the parameters examined for both the calf and the plantar muscles. Reduction in weight should thus be a first-line approach in preventing these changes that may significantly affect quality of life due to gait disturbances.

## Abstract (precis)

The present study adds to the literature of preclinical and human studies providing direct evidence between persistent obesity and changes in muscle architecture that affects joint loading.

## INTRODUCTION

Obesity is a world-wide health problem and is currently a pandemic not only restricted to western world, but also increasingly prevalent in eastern countries like China which traditionally was devoid of morphic changes associated with metabolic disease [1–3]. Obesity is strongly associated with musculoskeletal disorders of the lower limb [4]. Obese individuals show flat feet, reduced inversion-eversion range of motion and elevated peak plantar pressures when walking [4]. All of these results in unequal joint loading and force distributions, resulting in a vicious cycle of aberrant lower-extremity based weight-bearing [5–6]. After accounting for foot structure and walking speed, bodyweight has been reported to be significantly associated with elevated loading of the foot, particularly the forefoot and mid-foot [7–8]. These findings suggest that obesity predisposes to stresses to the foot via increased bodyweight and alterations to foot structure.

The pressure-related foot complaints also occur in older people, and causes enhanced defects in gait and mobility [9–10]. It has been studied that maximum forces and peak pressures under most regions of the foot could be mainly explained by differences in bodyweight, even in elderly populations [9]. The human foot is an efficient device and is an amazing, adaptive, powerful aid during walking, running, jumping and in locomotion up or down hill and on uneven ground [11]. Dysfunction of the foot however often arises from the foot losing its normal structural support, resulting in subtle changes in shape.

Obesity is associated with morphological and functional changes in the skeletal muscle including fibrosis. Studies suggest that macrophages in obese skeletal muscle may be altered to secrete transforming growth factor β1 (TGFβ1), a growth factor that can stimulate type I collagen gene expression via Smad3 activation [12]. Preclinical studies have indicated that high fat diet can increase skeletal muscle macrophage gene expression and fibrosis [13].

In the present study, we aimed to examine, using paired comparisons of subjects, of whether moderate duration of obesity in patients with mild to moderate elevations of body mass index (BMI), changes the fibrous contents of muscles that support the ankle mortices, namely calf and plantar muscles. MRI image segmentation and pixel correlations were used to analyse the changes. The present study attempted to examine whether architectural changes in the muscle mass changes in obesity of moderate duration, and whether such changes contribute to the destabilization of the ankle mortices.

## MATERIALS AND METHODS

### Study design

The recruitment of healthy and obese subjects occurred between the periods of 2013–2014. All subjects that participated in the final study provided written informed consent before enrolling in the study. The study protocol was performed only after receiving unobstructed approved by the Human Investigation Committee and performed in strict accordance with Helsinki guidelines. No subject had prior history of diabetes or tobacco smoking. Age-matched youth (25–35 years) and aged (55–65 years) (both male and female subjects) lean and obese subjects were included in the present study. Elderly subjects greater than 65 years old were not included in the present study to control for age-related sarcopenia. None of the patients had any alteration in range of motion for ankle dorsiflexion. Some elderly subjects had mild changes in foot shape. None of the subjects verbally reported that they had altered foot position (pronated or supinated) at rest position (and were also observed during the clinical examination). Though the obese subjects complained of occasional diffuse musculoskeletal pain, there was no evidence of any lower limb osteoarthritis. This was carefully assayed by examining bilateral limb radiographs prior to recruiting the subjects. Though restricting the groups of subjects led us to have control over the study design and did not allow variability in the complex presentations, however, the primary aim of the study was to obtain preliminary evidence if obesity existing for moderate duration impacted changes in the musculature that load the ankle and knee joints and whether there are changes in the histological architecture.

### MRI

Calf muscle and plantar imaging was performed using a 3T scanner. Sagittal T1 weighted and axial T2 weighted images were obtained. Images were backed up in a coded storage device and images analysed offline. All subjects were identified prior to the study. The analyses were performed in a blinded fashion.

### Image segmentation after volume reconstruction

Metamorph software was used to perform image segmentation analyses after 3D reconstruction of the images. Images were analysed to obtain the distinction between the boundaries of the anterolateral and posterior compartment muscles of both the legs. The plantar muscles were analysed as a composite. Images were segmented due to distinct contrast differences between fibrous and muscle tissues. Because of image heterogeneity, further analyses were performed to distinguish the differences in intra-muscle fibrosity (see below). No changes in γ settings were performed prior to analyses of images. All values were expressed as average of the bilateral parameters. Though there were slight differences in the parameters, especially in obese subjects, these values never obtained statistical significance.

The segmentation was performed based on homogeneity of the grey levels, which clustered based on the histologic characteristics. The software mainly used the Sobel edge detector for the determination of the transition zones [14]. The Sobel edge operator E is defined as a square matrix with triple dimensions such as: [1, 0, −1; 2, 0, −2; 1, 0, −1]. Convolution of the Sobel edge operator E with the image in the neighbourhood of a pixel manifests as vertical edge strength image [15]. This is referred to as edge co-occurrence matrix. The peak parameters along the leading diagonal were calculated using correlation techniques. The matrix is further labelled to present the content of the image. Labelling of the matrix necessitates elaboration about the distributions in the matrix. The labelled matrix is further used as a look-up table (LUT) for segmenting the regions of interest (ROIs) of an image and for evaluating the prominent edges for a particular edge operator. Using a combinatorial approach of several transforms, it is possible to detect edges of all orientations.

### GLCM and pixel entropy measurement

These set of analyses were performed using an operator developed with Matlab (Mathworks). Texture features were calculated from the statistical distribution of observed combinations of pixel intensities at specific positions in the images. According to the number of intensity points (pixels) for each combination, statistics may be classified into first-order, second-order and higher-order statistics. The grey level co-occurrence matrix (GLCM) method is a way of understanding the second order statistical texture features [16]. A GLCM is defined as a matrix where the number of rows and columns is same as the number of grey levels, *G*, in the image. The matrix element *P*(*i*, *j* | ∆*x*, ∆*y*) is defined as the relative frequency with which two pixels, separated by a pixel distance (∆*x*, ∆*y*), occur in the immediate vicinity, one with intensity *i* and the other with intensity *j*. The matrix element *P*(*i*, *j* | *d*, *θ*) contains the second order statistical probability values for differences between grey levels *i* and *j* at a given displacement distance *d* and at a particular angle (*θ*). Given an *M* × *N* neighbourhood of an input image with G grey levels from 0 to *G* − 1, let *f*(*m*, *n*) be the intensity at sample *m*, line *n* of the neighbourhood. Then, *P*(*i*, *j* | ∆*x*, ∆*y*)=*WQ*(*i*, *j* | ∆*x*, ∆*y*).

A number of texture features can be defined and derived from the GLCM. The following approaches were adapted: *G* is the number of grey levels used. *μ* is the mean value of *P*. *μx*, *μy*, *σx* and *σy* are the means and S.D. of *Px* and *Py*. Texture measures computed from GLCM-matrices: the marginal-probability matrix obtained by summing the rows of *P*(*i*, *j*): *Px*(*i*)=*G X* − 1 *j*=0 *P*(*i*, *j*) *Py*(*j*)=*G X* − 1 *i*=0 *P*(*i*, *j*).

Pixel entropy was used to define homogeneity in the imaged calf or plantar muscles. Entropy may be defined as: − *G X* − 1 *i*=0 *G X* − 1 *j*=0 *P*(*i*, *j*) × log(*P*(*i*, *j*)) [17]. Inhomogeneous pixel distributions have low first order entropy, whereas a homogeneous organization due to distinct separation between image components (due to homogeneity of tissue contents, for e.g., like the compacted fibrous tissue mass) has a high entropy.

Angular second moment (ASM): ASM=*G X* − 1 *i*=0 *G X* − 1 *j*=0 {*P*(*i*, *j*)}. ASM is an additional measure of homogeneity of an image [18]. A homogeneous digitized area is composed of only a few grey levels, giving a GLCM with only a few but relatively high values of *P*(*i*, *j*). Therefore, in a more contained image, the sum of squares will be high.

### Statistics

Data were expressed as means±S.E.M. Between-groups comparison between the different cohorts was performed with analyses of variance (ANOVA).

## RESULTS

### Enhanced ratio of fibrous to muscle mass during volumetric imaging of calf and plantar muscles

**Figure 1:**
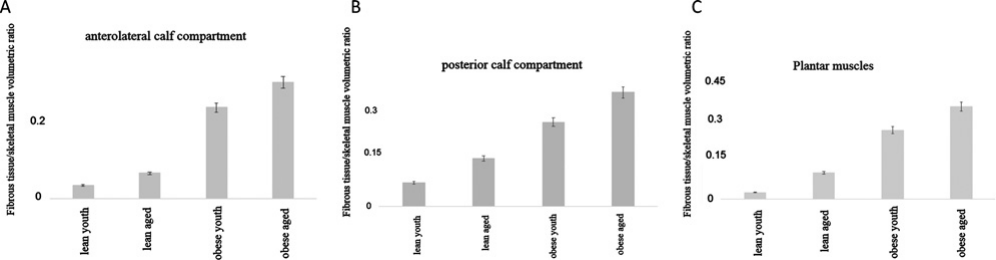
Bar histogram depicting increased fibrotic contents in comparison with muscle mass during volumetric MRI of anterolateral and posterior calf muscles and plantar muscles (**A**) These changes were examined in order to examine the hypothesis that total fibrous contents of muscles may change in obesity of moderate duration, which in turn may change the loading properties of the skeletal muscles and result in instability of the ankle joints. These computed differences in the means between groups were significant from control lean populations (*P*<0.0001, ANOVA). (**B**) The composite differences of all muscle groups were examined, including those of the posterior muscles. The differences in the means between groups were significant from control lean populations (*P*<0.0001, ANOVA). (**C**) The plantar muscles are the key supporting muscles of the inferior aspects of the ankle mortice. The differences in the means between groups were significant from control lean populations (*P*<0.0001, ANOVA).

In comparison with controls (lean youth and aged), fibrous tissue to muscle mass ratios were significantly elevated in both obese youth and elderly populace (*P*<0.0001, analyses of variance). These findings were true for all groups of muscles supporting the ankle mortice, viz. anterolateral and posterior group of calf muscles and superficial and deep plantar muscles. This observation was more pronounced in the posterolateral calf muscles and plantar muscles rather than the anterolateral group of calf muscles. The means of the ratios for the anterolateral group of calf muscle were as follows: 0.03±0.004 versus 0.06±0.0104 versus 0.23±0.0071 versus 0.3±0.006 (mean squares, 0.4089, *F*=308.8, *P*<0.0001, ANOVA) (Figure 1A). The means of the ratios for the posterior group of calf muscle were as follows: 0.06±0.003 versus 0.13±0.003 versus 0.23±0.003 versus 0.31±0.004 (mean squares, 0.2989, *F*=818.4, *P*<0.0001, ANOVA) (Figure 1B). The means of the ratios for the plantar muscles were as follows: 0.02±0.002 versus 0.09±0.017 versus 0.244±0.006 versus 0.33±0.007 (mean squares, 0.4782, *F*=190.8, *P*<0.0001, ANOVA) (Figure 1C).

### Grey level co-occurrence matrix correlation and entropy analyses in posterior group of calf muscle and plantar muscles

In order to confirm that the increased fibrosity as assessed by image segmentation of MRI images is a true observation and given the heterogeneity in muscle mass due to numerous cellular components, we performed additional analyses of correlation of the pixels in the obtained images. GLCMs were observed, in addition to the chaos between the imaged segments. This last parameter was assayed by documenting the entropy between pixels. The orientation of the pixels was also observed by the angular moments, the changes with imaging in obese subjects coincided with the GLCM and entropy (results not shown).

**Figure 2:**
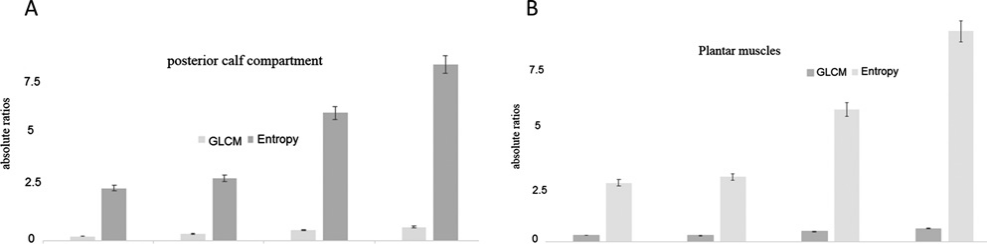
Pixel correlation (GLCM) and enhanced pixel entropy in different muscles in youth and aged obese subjects (**A**) Reduced pixel correlation (GLCM) and enhanced pixel entropy in posterior calf muscles in youth and aged obese subjects. The uniformity in appearance or non-uniformity in obesity of some duration was examined by quantitative aspects of the correlation of appearances of individual pixels after obtaining MRI images of calf and plantar muscles. Even if quantitative measures are not obtained, these pixel correlations may be viewed to predict ongoing instability and can form semi-quantitative guidelines for management including advice for weight reduction as well as use of orthotic devices. The differences in the means (for both entropy and GLCM) between groups were significant from control lean populations (*P*<0.0001, ANOVA). (**B**) Diminished pixel correlation (GLCM) and enhanced pixel entropy in plantar muscles in youth and aged obese subjects. The differences in the means between groups (both GLCM and entropy) were significant from control lean populations (*P*<0.0001, ANOVA).

The GLCMs in the different groups were as follows for the posterior group of calf muscles: 0.2±0.001 versus 0.32±0.033 versus 0.49±0.061 versus 0.61±0.072 (*P*<0.001, ANOVA). The entropy values were as follows for the posterior group of calf muscles: 2.34±0.41 versus 2.76±1.001 versus 5.67±0.86 versus 7.81±1.32 (*P*<0.001, ANOVA) (Figure 2A).

The GLCMs in the different groups were as follows for the plantar muscles: 0.28±0.01 versus 0.27±0.04 versus 0.44±0.056 versus 0.57±0.01 (*P*<0.001, ANOVA). The entropy values were as follows for the posterior group of calf muscles: 2.53±0.201 versus 2.78±0.34 versus 5.67±1.4561 versus 9.01±1.84 (*P*<0.001, ANOVA) (Figure 2B).

## DISCUSSION

The results of the present study are indicative, pending additional validation studies in larger samples, that there is persistent mechanical strain that causes progressive damage to myogenic architecture in obese subjects. This happens irrespective of the age distribution, at least within the context of the current study where we excluded patients older than 65 years age. In the current study, we incorporated subjects who were obese for at least 2 years or more; due to lack of controls for age-based distribution, our data do not provide insights into the duration of obesity on fibrotic changes in muscle architecture.

Recently, studies have shown that fat infiltration occurs in the calf muscles of aged populations [19]. These studies do not comment on the contents of the fibrotic tissues *per se*. Numerous recent studies have shown that defects in joint loading mechanisms results in pro-fibrotic reactions, which further causes strain on the joints [20–25]. None of our obese subjects had any evidence of any lower extremity fracture. However, the nature of the fibrosis indicates that there is considerable strain on the posterior and plantar calf muscles, probably due to unequal distribution of weight during supine and upright positions of the subjects. Though the subjects had no evidence of fracture, the nature and distribution of the muscle fibrosis indicates that there might be a combination of stress related to performance of pronation-supination and external/internal rotation of the ankle mortices. Though we have observed only static measurements of the state of the muscle/fibrous tissue ratios, it is likely that these changes might cause stress on the functions of all the joints of the lower extremities, viz. ankle mortice, knee and hip joints respectively.

Preliminary studies have suggested that plantar heel pain is associated with higher BMI and reductions in some foot and ankle strength and flexibility measures [7]. Although these factors could not be specifically correlated and resulted from either causal reasons or consequential, these factors were all potentially modifiable and could be targeted in the management of plantar heel pain. The plantar heel pain was always reported in the group with a higher BMI, diminution in ankle dorsiflexion range of motion, reduced ankle evertor and toe flexor strength and also inverted inversion/eversion strength ratio [7]. These studies revealed that there were no differences between groups for foot alignment, muscle strength including dorsiflexor or invertor strength, ankle inversion or eversion range of motion, generalized hypermobility, occupational standing time or exercise level [7,25]. These correlations may be made with controlled studies in future.

The current study demonstrates significant changes in lower extremity muscle fibrotic contents in obese subjects independent of age, even though our subject series had moderate elevations of BMI and only for approximately 2 years. Pharmacologic modulation may be explored to decrease fibrosis and prevent age related muscle changes, thus helping to transmit body weight efficiently through the ankle joints. The present study is a significant attempt to the scant literature of preclinical and human studies providing direct evidence to the link between persistent obesity and changes in muscle architecture and affecting the joint loading [26–28]. Based on the present study, weight loss may be encouraged to improve the muscle changes with sustained obesity [29–30].

## Abbreviations

ASM: angular second moment
BMI: body mass index
GLCM: grey level co-occurrence matrix
LUT: look-up table
ROIs: regions of interest
TGFβ1: transforming growth factor β1
